# Indirect interactions involving the PsbM or PsbT subunits and the PsbO, PsbU and PsbV proteins stabilize assembly and activity of Photosystem II in *Synechocystis* sp. PCC 6803

**DOI:** 10.1007/s11120-024-01091-9

**Published:** 2024-03-15

**Authors:** Faiza Arshad, Julian J. Eaton-Rye

**Affiliations:** Department of Biochemistry, University of Otago, Dunedin, New Zealand

**Keywords:** Photosynthesis, Photosystem II, Biogenesis, Lumenal proteins, Transmembrane proteins, PsbO, PsbU, PsbV, PsbT, PsbM

## Abstract

The low-molecular-weight PsbM and PsbT proteins of Photosystem II (PS II) are both located at the monomer-monomer interface of the mature PS II dimer. Since the extrinsic proteins are associated with the final step of assembly of an active PS II monomer and, in the case of PsbO, are known to impact the stability of the PS II dimer, we have investigated the potential cooperativity between the PsbM and PsbT subunits and the PsbO, PsbU and PsbV extrinsic proteins. Blue-native polyacrylamide electrophoresis and western blotting detected stable PS II monomers in the ∆PsbM:∆PsbO and ∆PsbT:∆PsbO mutants that retained sufficient oxygen-evolving activity to support reduced photoautotrophic growth. In contrast, the ∆PsbM:∆PsbU and ∆PsbT:∆PsbU mutants assembled dimeric PS II at levels comparable to wild type and supported photoautotrophic growth at rates similar to those obtained with the corresponding ∆PsbM and ∆PsbT cells. Removal of PsbV was more detrimental than removal of PsbO. Only limited levels of dimeric PS II were observed in the ∆PsbM:∆PsbV mutant and the overall reduced level of assembled PS II in this mutant resulted in diminished rates of photoautotrophic growth and PS II activity below those obtained in the ∆PsbM:∆PsbO and ∆PsbT:∆PsbO strains. In addition, the ∆PsbT:∆PsbV mutant did not assemble active PS II centers although inactive monomers could be detected. The inability of the ∆PsbT:∆PsbV mutant to grow photoautotrophically, or to evolve oxygen, suggested a stable oxygen-evolving complex could not assemble in this mutant.

**Supplementary Information:**

The online version contains supplementary material available at 10.1007/s11120-024-01091-9.

## Introduction

Photosystem II (PS II) catalyzes the oxidation of water and reduction of plastoquinone in all oxygenic photosynthetic organisms (Vinyard et al. 2013). Cyanobacteria have been used as model systems to study the water-splitting reaction and this has led to high-resolution structures (< 2.0 Å or similar) being obtained from *Thermosynechococcus vulcanus*, *T. elongatus* and *Synechocystis* sp. PCC 6803 (Umena et al. 2011; Kern et al. 2018; Gisriel et al. 2022). These structural studies have confirmed PS II is present as a dimer of approximately 700 kDa (Shen 2015).

Each PS II monomer contains two reaction center proteins, D1 (PsbA) and D2 (PsbD), that bind most of the redox active cofactors (Cardona et al. 2012). Flanking D1 and D2 are two chlorophyll *a*-containing core antenna proteins, CP43 (PsbC) and CP47 (PsbB) (Müh and Zouni 2020). In cyanobacteria the PS II complex also contains 13 low-molecular-weight (LMW) proteins that typically span the membrane once (PsbZ has two membrane-spanning helices) and three hydrophilic proteins that attach to the lumenal side of PS II known as PsbO, PsbU and PsbV (Shi et al. 2012; Bricker et al. 2012; Roose et al. 2016). In *Synechocystis* sp. PCC 6803 a fourth hydrophilic protein, PsbQ (or CyanoQ) has been detected but this subunit was not found in the PS II structures from *T. vulcanus* or *T. elongatus* (Shen 2015; Giriel et al. 2022).

Biogenesis of PS II likely proceeds through a series of steps where the mature complex is built up from pre-assembly modules involving D1, D2, CP43 and CP47 (Komenda et al. 2012). In this model the D1 and D2 pre-assembly modules combine to form the reaction center complex. Subsequently the CP47 pre-assembly module associates with the reaction center, forming the RC47 complex, and this is followed by the association of the CP43 module (Nickelsen and Rengstl 2013; Johnson and Pakrasi 2022). At the final stage of assembly, the Mn_4_O_5_Ca oxygen-evolving complex (OEC) is incorporated along with the binding of the PsbO, PsbU and PsbV extrinsic proteins before the complex transitions into its final dimeric form (Bao and Burnap 2016).

The reaction center and RC47 complexes, as well as each pre-assembly module, are associated with specific assembly factors that are not retained in the mature complex as well as a subset of the LMW proteins (Nickelsen and Rengstl 2013). Among the LMW proteins, PsbL (~ 4.5 kDa), PsbM (~ 3.9 kDa) and PsbT (~ 3.5 kDa) are found as part of the RC47 pre-complex and are located at the monomer-monomer interface in the final complex (Umena et al. 2011; Boehm et al. 2012). Moreover, PsbL and PsbT have been detected in the CP47 assembly module indicating they bind before the formation of the RC47 complex (Boehm et al. 2011). Deletion of PsbL in *Synechocystis* sp. PCC 6803 arrested PS II assembly resulting in accumulation of the RC47 intermediate (Anbudurai and Pakrasi 1993; Bentley et al. 2008); however, the ∆PsbM and ∆PsbT strains, as well as the ∆PsbM:∆PsbT mutant, remained photoautotrophic (Bentley et al. 2008).

At the monomer-monomer interface both PsbM subunits appear to stabilize the dimer through a putative leucine zipper, although PS II dimers do form in the absence of PsbM (Bentley et al. 2008; Kawakami et al. 2011; Uto et al. 2017). Additionally, but to a lesser extent than removal of PsbM, deletion of PsbT also destabilizes the dimer (Bentley et al. 2008; Fagerlund et al. 2021; Forsman and Eaton-Rye 2021). Furthermore, removal of either PsbM or PsbT impaired electron transfer between the primary and secondary plastoquinone electron acceptors (Q_A_ and Q_B_, respectively) in the Q_A_-Fe-Q_B_ complex responsible for electron transfer from PS II into the plastoquinone pool in the thylakoid membrane (Müh et al. 2012; Uto et al. 2017; Fagerlund et al. 2020). The non-heme iron in the Q_A_-Fe-Q_B_ complex is coordinated by histidine ligands from D1 and D2 and a bicarbonate ion (Umena et al. 2011; Shevela et al. 2012). Notably, the deletion of either PsbM or PsbT perturbed the bicarbonate-binding environment providing an explanation for why electron transfer between Q_A_ and Q_B_ is impacted (Uto et al. 2017; Forsman et al. 2020; Forsman and Eaton-Rye 2021). The absence of either PsbM or PsbT also increased the susceptibility of PS II to photodamage under high light (Biswas and Eaton-Rye 2018; Fagerlund et al. 2020).

The stability of the PS II dimer is also dependent on the PsbO protein, and to a lesser extent, the PsbV subunit (Bentley and Eaton-Rye 2008; Komenda et al. 2010). PsbO stabilizes the dimer through binding to lumenal domains of CP47 on the same and adjacent monomers (De Las Rivas and Barber 2004). While both the ∆PsbO and ∆PsbV strains are photoautotrophic (reviewed in Bricker et al. 2012), the ∆PsbV mutant in *T. vulcanus* accumulated a PS II dimer with a bound Psb27 assembly factor (Huang et al. 2021). The Psb27 assembly factor needs to be released upon conversion of the RC47 complex to the active PS II monomer concomitantly with the binding of the CP43 assembly module (Zabret et al. 2021). This step of PS II biogenesis completes the formation of the Q_A_-Fe-Q_B_ complex and facilitates binding of the OEC and the PsbO, PsbU and PsbV subunits (Zabret et al. 2021; Xiao et al. 2021). This last assembly step before dimerization also appears to be accompanied by the binding of the PsbJ LMW protein. Unlike their corresponding single deletion mutants the ∆PsbJ:∆PsbO and ∆PsbJ:∆PsbV strains, constructed in *Synechocystis* sp. PCC 6803, were not photoautotrophic suggesting an intact OEC could not form in these mutants (Choo et al. 2022). Given the combined effect of removing PsbJ in combination with PsbO and PsbV, and the requirement for PsbM and PsbT to be present at the monomer-monomer interface for dimer stability, we have investigated the effect of removing the extrinsic proteins in combination with deletion of PsbM or PsbT and characterized the impact of the double deletions on PS II assembly and activity.

## Materials and methods

### Strains and growth conditions

The *Synechocystis* sp. PCC 6803 wild type used in this study was the glucose tolerant strain GT-O1 (Williams 1988; Morris et al. 2014). All mutants were constructed in GT-O1 cells as described in Eaton-Rye (2011). The *psbT* gene was deleted by replacing the sequence from 143 bp upstream of the start codon to 86 bp downstream of the gene with a 1.1 kb chloramphenicol-resistance cassette. The *psbM* gene was also deleted between nucleotides 4 and 108 by inserting a 1.2 kb kanamycin-resistance cassette in the ∆PsbM mutant and the same region of *psbM* was replaced by a 2 kb spectinomycin-resistance cassette in the ∆PsbM:∆PsbV and ∆PsbM:∆PsbU double mutants. The ∆PsbO strain was constructed by deleting the region between nucleotides 467 and 555 with the kanamycin-resistance cassette in the ∆PsbO mutant and by a spectinomycin-resistance cassette in the ∆PsbM:∆PsbO and ∆PsbT:∆PsbO strains. The *psbU* and *psbV* genes were inactivated by insertion of the kanamycin-resistance cassette. In the case of *psbU* the resistance cassette was inserted between nucleotides 154 and 376 and in the case of *psbV*, between nucleotides 14 and 120. All mutants were confirmed by Sanger sequencing with segregation confirmed by PCR (Fig. S1 and Table S1).

Strains were maintained on BG-11 media plates containing 5 mM glucose, 20 µM atrazine, 10 mM TES-NaOH (pH 8.2) and 0.3% sodium thiosulfate along with appropriate antibiotics. Kanamycin and spectinomycin were present at 25 µg mL^-1^ and chloramphenicol was used at 15 µg mL^-1^. Liquid cultures for physiological characterization were initially grown mixotrophically to mid-logarithmic phase at 30 °C under continuous illumination at 30 µmol photons m^-2^ s^-1^ in BG-11 medium containing 5 mM glucose and the relevant antibiotics (Eaton-Rye 2011).

### Photoautotrophic growth

Liquid cultures, prepared as described above, were harvested by centrifugation at 5000×*g* for 10 min at room temperature and resuspended in unbuffered BG-11 at an optical density (OD) of 0.05 at 730 nm. Cells were then grown photoautotrophically at 30 °C under continuous illumination at 30 µmol photons m^-2^ s^-1^ and the OD_730 nm_ recorded at 24 h intervals for 168 h with an Evolution 201 UV/Vis spectrophotometer (Thermo Fisher Scientific, Waltham, MA, U.S.A.).

### Oxygen evolution

Cultures were grown mixotrophically and resuspended in BG-11 containing 25 mM HEPES-NaOH (pH 7.5) at the chlorophyll *a* concentration of 10 µg mL^− 1^. The concentration of chlorophyll *a* was determined according to MacKinney (1941). The cells were incubated for 30 min at 30 ^o^C at 30 µmol photons m^− 2^ s^− 1^ on a rotary shaker at 100 rpm. Oxygen evolution was measured using a Clark-type oxygen electrode (Hansatech, King’s Lynn, U.K.) at 30 ^o^C. Saturating light (2 mmol photons m^− 2^ s^− 1^) was provided by an FLS1 light source (Hansatech) passed through a Melis Griot OG 590 sharp cutoff red glass filter. Photosystem II-specific oxygen evolution was measured in the presence of 0.2 mM 2,5-dimethyl-1,4-benzoquinone (DMBQ) or 2,5-dichloro-1,4-benzoquinone (DCBQ) (both in the presence of 1 mM K_3_Fe(CN)_6_). Oxygen evolution resulting from whole chain electron transport was measured in the presence of of 15 mM NaHCO_3_.

### *Variable chlorophyll a* fluorescence induction and decay

Cells were grown mixotrophically as described above and resuspended at 5 µg mL^− 1^ chlorophyll *a* in BG-11 containing 25 mM HEPES-NaOH (pH 7.5). The cells were then incubated as described for the oxygen-evolution assays before being dark adapted for 5 min prior to the measurement. An FL-3500 dual-modulation kinetic fluorometer (PSI Instruments, Brno, Czech Republic) was used to measure the fluorescence induction traces. To determine the minimum level of fluorescence (Fo), cells were first subjected to four blue (455 nm) measuring flashes spaced at 200 µs and then illuminated with blue (455 nm) actinic light over 10 s at room temperature. The variable fluorescence was detected using 455 nm measuring flashes. The actinic light voltage was set to 50% and the measuring light voltage to 80%. The same instrument was used to determine the fluorescence decay kinetics. Dark-adapted cells were exposed to four measuring light pulses to determine Fo and then given a single 30 µs saturating actinic flash (455 nm). The fluorescence decay was obtained by giving a series of measuring flashes starting 26 µs after the actinic flash. The actinic light voltage was set to 100% and the measuring light voltage was set to 80%. When present, 3-(3,4-dichlorophenyl)-1,1-dimethylurea (DCMU) was added to the final concentration of 40 µM at 1 min before the measurement. The fluorescence decay kinetics were analyzed by the fitting equations provided by Vass et al. (1999).

### Low-temperature chlorophyll *a* fluorescence emission spectroscopy

Cultures were prepared as described above for the measurements of variable chlorophyll *a* fluorescence. After incubation, 0.5 mL of the culture was snap frozen in liquid nitrogen. Emission spectra at 77 K were recorded from 600 to 780 nm after exciting the cells with wavelengths of 440 or 580 nm using a modified MPF-3 L spectrophotometer (PerkinElmer, Waltham, MA, U.S.A.) as described in Jackson et al. (2014). All spectra were normalized to the Photosystem I (PS I) emission at 725 nm.

### Thylakoid isolation

Cultures were grown mixotrophically and washed twice in 50 mM HEPES-NaOH (pH 7.5), 20 mM CaCl_2_, 10 mM MgCl_2_, 1 mM ε-aminocaproic acid, 1 mM phenylmethylsulfonyl fluoride (PMSF) and 2 mM benzamidine. The cells were re-suspended in 50 mM HEPES-NaOH (pH 7.5), 20 mM CaCl_2_, 10 mM of MgCl_2_, 800 mM sorbitol, 1 M betaine monohydrate, 1 mM ε-aminocaproic acid, 1 mM PMSF and 2 mM of benzamidine (disruption buffer). The cells were then broken using a bead-beater (Biospec Products, Bartlesville, OK, U.S.A.) in the presence of an equal volume of 0.1 mm zirconia beads using five 20 s cycles at 4800 rpm at 4 ^o^C, keeping the sample on ice for 30 s between each cycle. Thylakoid membranes were separated from beads and cell debris at 8000×*g* for 10 min and collected by centrifugation at 25,000×*g* for 1 h. The pellet was then re-suspended in 8 mL of disruption buffer and collected at 25,000×*g* for 30 min. The isolated thylakoids were re-suspended in 25 mM of Bis-Tris HCl (pH 7.0), 20% w/v glycerol and 20 µM Pefabloc (Sigma-Aldrich, St Louis, MO, U.S.A.) and stored at -80 ^o^C.

### Gel electrophoresis and western blotting

Extracted thylakoids at 0.5 µg mL^− 1^ chlorophyll *a* were solubilized in the presence of 3% n-dodecyl-ß-D-maltoside in buffer containing 25 mM of Bis-Tris HCl (pH 7.0), 20% w/v glycerol and 20 µM Pefabloc (Sigma-Aldrich). The protein complexes were separated by blue native polyacrylamide gel electrophoresis (BN-PAGE) on a linear 3 – 12% gradient polyacrylamide gel at 4 ^o^C. The electrophoresis was carried out using a Protean II cell (Bio-Rad Laboratories, Hercules, CA, U.S.A.) at 150 V for 8 h (Jackson et al. 2014). Sodium dodecyl sulfate (SDS) PAGE was carried out on 15% polyacrylamide gels. Thylakoids at 10 µg mL^− 1^ chlorophyll *a* in 0.5 M Tris-HCl (pH 6.8), 10% SDS, 10% v/v glycerol, 5% 2-mercaptoethanol and 1% w/v bromophenol blue were heated for 10 min at 65 ^o^C and the electrophoresis run in a Protean II cell (Bio-Rad) at 200 V. After completion of the runs, the BN-PAGE gels were blotted onto polyvinylidene difluoride membrane and SDS-PAGE gels onto nitrocellulose membrane. The membranes were probed with antibodies specific to the PS II proteins D1, D2, CP43 and PsbO (Agrisera, Vännäs, Sweden) and PsbV (PhytoAB, San Jose, CA, U.S.A.). Specific bands were detected by enhanced chemiluminescence using an Odyssey Fc Imaging System (LI-COR Biotechnology, Lincoln, NE, U.S.A.).

## Results

### Removal of the extrinsic proteins in cells lacking PsbM and PsbT impaired photoautotrophic growth and assembly

**Fig. 1:**
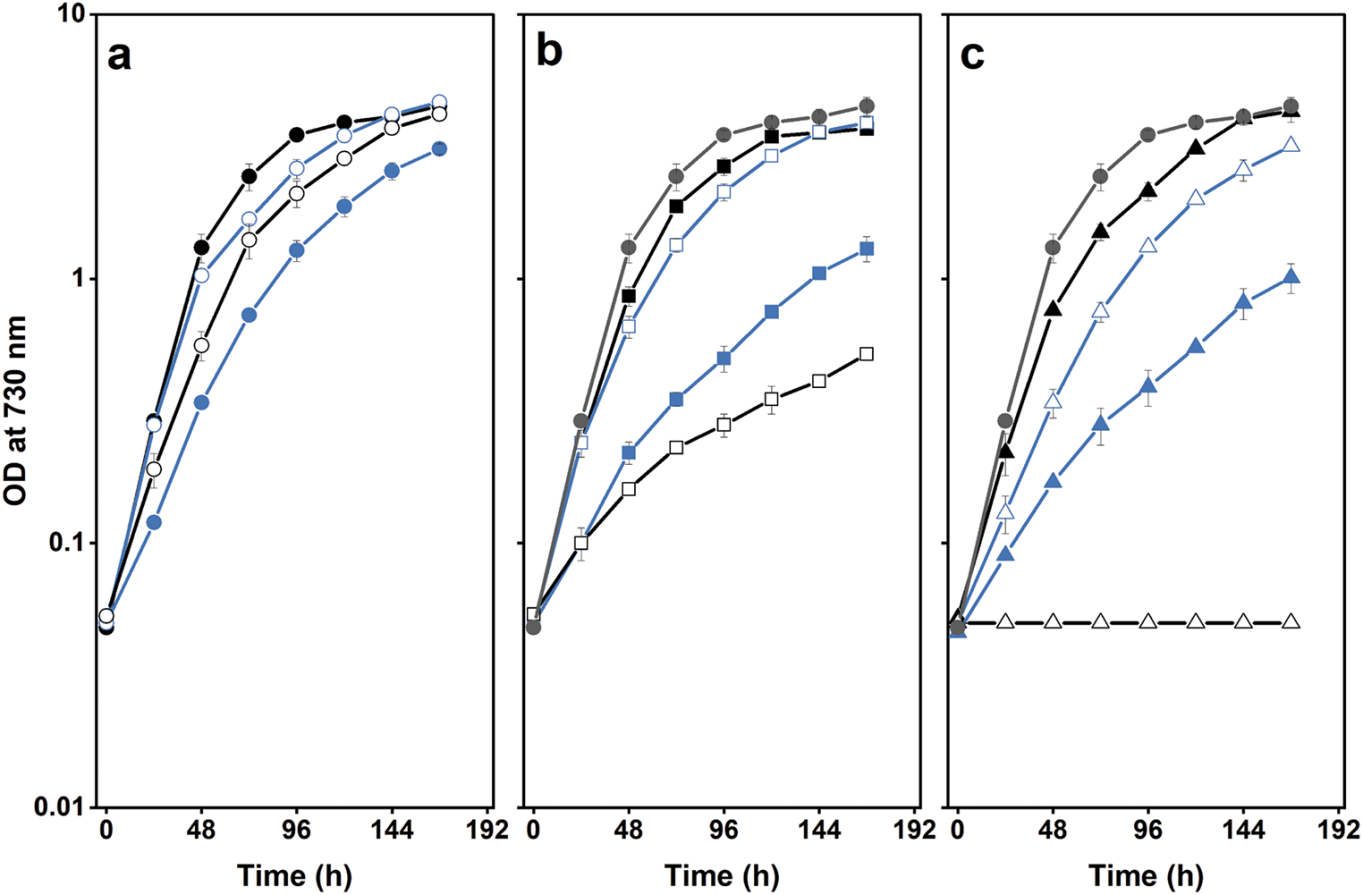
Photoautotrophic growth of strains monitored by the optical density at 730 nm. a Wild type (black filled circles), ΔPsbO (blue filled circles), ΔPsbU (blue empty circles) and ΔPsbV (black empty circles). **b** ΔPsbM (black filled squares), ΔPsbM:ΔPsbO (blue filled squares), ΔPsbM:ΔPsbU (blue empty squares), and ΔPsbM:ΔPsbV (black empty squares). **c** ΔPsbT (black filled triangles), ΔPsbT:ΔPsbO (blue filled triangles), ΔPsbT:ΔPsbU (blue empty triangles) and ΔPsbT:ΔPsbV (black empty triangles). In panels **b** and **c** the control data are repeated in grey to aid in comparing the different growth rates. Error bars represent the standard error for three independent experiments; error bars smaller than the symbols are not shown

The impact of removing the PsbO, PsbU and PsbV proteins in strains lacking either PsbM or PsbT was initially evaluated by comparing photoautotrophic growth between the mutants (Fig. 1). In agreement with previous studies, removal of the extrinsic proteins in the wild-type background did not prevent growth (Fig. 1a) (reviewed in Bricker et al. 2012). The doubling times for the wild type and the ∆PsbU strain were similar (~ 12 h), although the growth of ∆PsbU cells slowed after 48 h. In addition, the doubling time was extended to 16 h for ∆PsbV cells and ~ 19 h for the ∆PsbO mutant. Also, as previously observed, the photoautotrophic growth rates of ∆PsbM and ∆PsbT cells were similar to wild type with a doubling time of ~ 14 h (Fig. 1b, c) (Biswas and Eaton-Rye 2018; Fagerlund et al. 2020).

The effect of removing the extrinsic proteins in the ∆PsbM and ∆PsbT backgrounds is shown in Fig. 1b, c. The doubling time was approximately 15 and 19 h in the ∆PsbM:∆PsbU and ∆PsbT:∆PsbU strains, respectively, and increased to ~ 24 h in ∆PsbM:∆PsbO cells and to ~ 29 h in ∆PsbM:∆PsbV and ∆PsbT:∆PsbO cells (Fig. 1b, c). The ∆PsbT:∆PsbV strain, however, was not photoautotrophic (Fig. 1c).

**Fig. 2:**
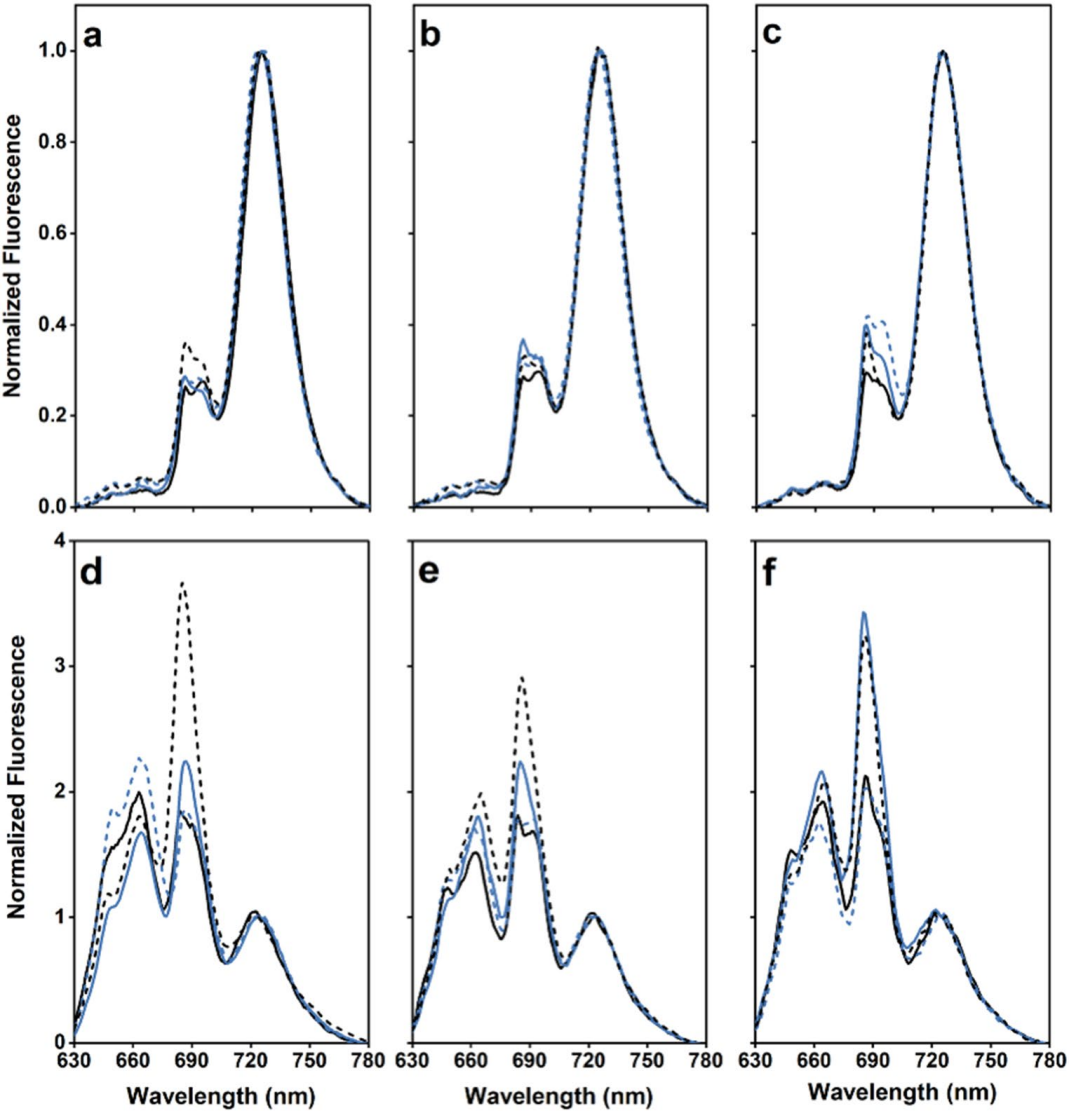
Low temperature (77 K) fluorescence emission spectra. The cells were excited at 440 nm in panels (**a – c**) and 580 nm in panel (**d – f**). **a, d** Wild type (black line), ΔPsbO (blue line), ΔPsbU (blue dashed line) and ΔPsbV (black dashed line). **b, e** ΔPsbM (black line), ΔPsbM:ΔPsbO (blue line), ΔPsbM:ΔPsbU (blue dashed line) and ΔPsbM:ΔPsbV (black dashed line). **c, f** ΔPsbT (black line), ΔPsbT:ΔPsbO (blue line), ΔPsbT:ΔPsbU (blue dashed line) and ΔPsbT:ΔPsbV (black dashed line). Spectra are the average of three biological repeats and are normalized to the fluorescence emission from PS I at 725 nm

Low-temperature (77 K) fluorescence emission spectra, excited at 440 nm, were collected for all strains to provide an indication of the relative level of assembled and unassembled PS II complexes (Lamb et al. 2018). The emission at 685 nm arises from PS II pre-assembly complexes and modules, and from CP43 in mature PS II; the emission at 695 nm arises from CP47 in the mature complex (Boehm et al. 2011). All spectra were normalized to the emission at 725 nm arising from PS I. The intensity of the PS II-specific bands at 685 and 695 nm were similar in the wild type and the ∆PsbU strain while the 685 nm emission was slightly elevated in the ∆PsbO mutant and both the 685 and 695 nm bands were increased in the ∆PsbV strain (Fig. 2a). Additionally, the ∆PsbM strain exhibited a similar emission spectrum to wild type; however, the 685 nm emission was elevated in the ∆PsbT strain (Fig. 2b, c) and is consistent with Forsman and Eaton-Rye (2021).

With the double mutants, an elevated emission at 685 nm compared to 695 nm was observed in the spectra of the ∆PsbM:∆PsbO and ∆PsbT:∆PsbO strains (Fig. 2b, c). Furthermore, the amplitude of the PS II-specific peaks in the ∆PsbM:∆PsbU and ∆PsbM:∆PsbV mutants were elevated and this was more pronounced in the ∆PsbT:∆PsbU strain (Fig. 2b, c). An elevated emission at 685 nm was also present in the ∆PsbT:∆PsbV cells.

For each strain the low-temperature fluorescence emission spectra were also measured after excitation with 580 nm light which preferentially excites the phycobilisome antenna (Fig. 2d–f). An increased emission at 685 nm was observed in all mutants where PsbV was absent. In addition, elevated emission at 685 nm was also observed in the absence of PsbO; in the case of the ∆PsbT:∆PsbO cells, the 685 nm emission was similar to that obtained with the ∆PsbT:∆PsbV mutant.

### Impaired PS II assembly as detected by Western blotting

**Fig. 3:**
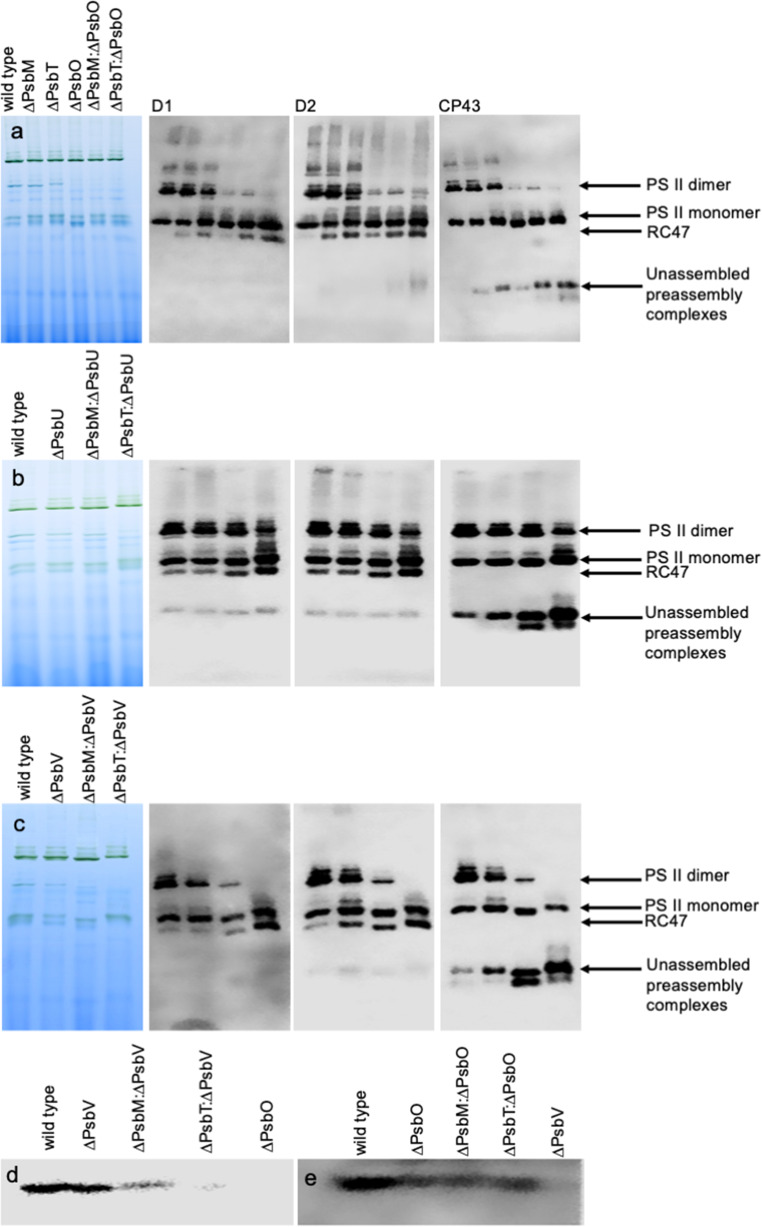
Analysis of PS II assembly by BN-PAGE followed by western blotting using antibodies against the D1, D2 and CP43 proteins (**a – c**) and SDS-PAGE followed by western blotting using antibodies against the PsbO and PsbV proteins (**d** and **e**). **a** Lanes 1 to 6: wild type, ∆PsbM, ∆PsbT, ∆PsbO, ∆PsbM:∆PsbO ∆PsbT:∆PsbO. **b** Lanes 1 to 4: wild type, ∆PsbU, ∆PsbM:∆PsbU and ∆PsbT:∆PsbU. **c** Lanes 1 to 4: wild type, ∆PsbV, ∆PsbM:∆PsbV and ∆PsbT:∆PsbV. **d** Western blot of strains lacking the PsbV protein and probed with an antibody for PsbO. The ∆PsbO mutant was included as a control. **e** Western blot of strains lacking the PsbO protein and probed with an antibody for PsbV. The ∆PsbV mutant was included as a control. The experiments were repeated three times with similar results

The assembly of PS II was also investigated using BN-PAGE followed by western blots probed with antibodies to D1, D2 and CP43. In Fig. 3a the impact of removing PsbO was evaluated. While the ∆PsbM and ∆PsbT strains both exhibited monomer and dimer bands at similar levels to those obtained with wild type, in all strains lacking the PsbO protein the amount of dimer was considerably reduced. In addition, in cells lacking PsbT, an additional band just below the dimer was observed with the D1 and D2 antibodies that was not detected with the CP43 antibody, consistent with an earlier report (Bentley et al. 2008). All mutants also accumulated more RC47 complex than wild type with the most prominent signal for the RC47 complex observed in the ∆PsbT:∆PsbO strain (Fig. 3a).

Removal of PsbU had little impact on the relative levels of monomers and dimers although the ∆PsbM:∆PsbU and ∆PsbT:∆PsbU strains had elevated levels of a putative pre-complex (or module) containing CP43 (Fig. 3b). A similar detection of CP43-containing bands was observed with the ∆PsbM:∆PsbV and ∆PsbT:∆PsbV strains (Fig. 3c). Moreover, the dimer level was reduced in the ∆PsbM:∆PsbV mutant and absent from the lane corresponding to the ∆PsbT:∆PsbV strain (Fig. 3c). The lane corresponding to the ∆PsbT:∆PsbV mutant also ran with a gel shift consistent with the presence of carotenoids resulting from a stress response in these cells (Choo et al. 2022). Interestingly, SDS-PAGE followed by western blotting revealed that the ∆PsbM:∆PsbV and ∆PsbT:∆PsbV strains bound PsbO weakly with almost no PsbO detected in the ∆PsbT:∆PsbV mutant. In contrast, the double mutants lacking PsbO were still able to bind PsbV (Fig. 3d, e).

### Photosystem II activity as determined by measurements of oxygen evolution

**Fig. 4:**
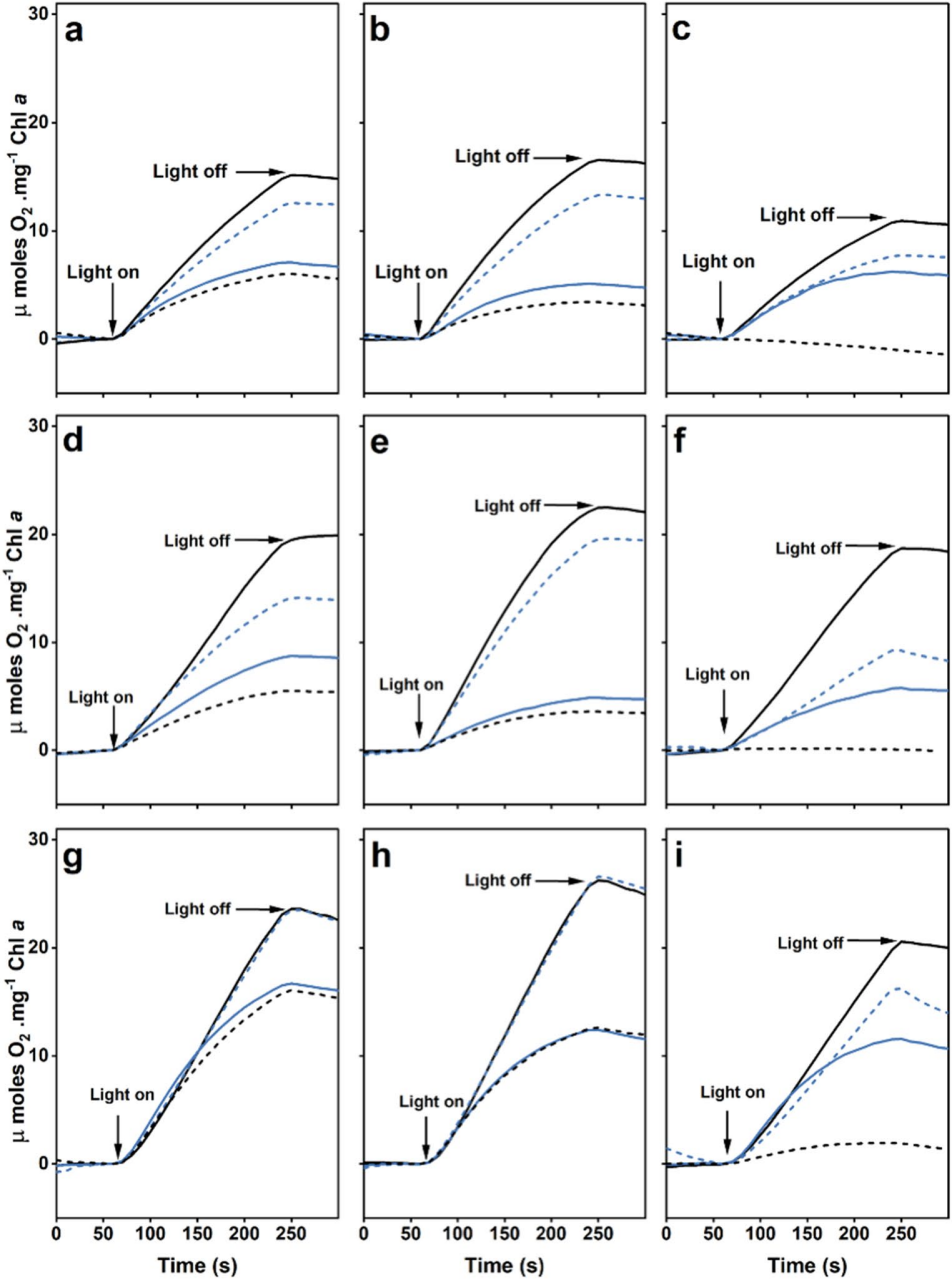
Oxygen evolution supported by PS II-specific electron acceptors or whole chain electron transport in the presence of sodium bicarbonate. **a – c** Oxygen evolution supported by DMBQ. **d – f** Oxygen evolution supported by DCBQ. **g – i** Oxygen evolution supported by NaHCO_3_. In panels **a**, **d**, **g** the traces are: wild type (black solid line), ΔPsbO (blue solid line), ΔPsbU (blue dashed line), ΔPsbV (black dashed line); in panels **b**, **e**, **h** the traces are: ΔPsbM (black solid line), ΔPsbM:ΔPsbO (blue solid line), ΔPsbM:ΔPsbU (blue dashed line), ΔPsbM:ΔPsbV (black dashed line), and in panels **c**, **f**, **i** the traces are: ΔPsbT (black solid line), ΔPsbT:ΔPsbO (blue solid line), ΔPsbT:ΔPsbU (blue dashed line), ΔPsbT:ΔPsbV (black dashed line). Each trace represents the average of three independent experiments

To investigate the activity of PS II in the different strains, oxygen evolution was measured using the PS II-specific electron acceptors DMBQ and DCBQ; in addition, whole chain electron transport was determined in the presence of bicarbonate (Table 1). For each electron acceptor the rates obtained for the single mutants were similar to published results (Biswas and Eaton-Rye 2018; Fagerlund et al. 2020; Forsman and Eaton-Rye 2021; Choo et al. 2022). The removal of PsbU had little impact in the ∆PsbM:∆PsbU strain in the presence of either DMBQ or DCBQ; however, the ∆PsbT:∆PsbU strain was impaired to a greater extent in the presence of DCBQ than DMBQ (Table 1; Fig. 4). The removal of PsbO in the ∆PsbO strain reduced the rate of oxygen evolution by ~ 40% in the presence of DMBQ and ~ 55% in the presence of DCBQ and these rates declined further in the ∆PsbM:∆PsbO and ∆PsbT:∆PsbO strains. Similarly, the removal of PsbV in the ∆PsbV and ∆PsbM:∆PsbV strains resulted in reduced rates for both PS II-specific reactions; however, no oxygen evolution was detected in the ∆PsbT:∆PsbV mutant with any of the electron acceptors. In contrast, the rates obtained for whole chain electron transport in the different strains were typically stimulated, with the ∆PsbO, ∆PsbU and ∆PsbV strains exhibiting initial rates similar to wild type. Compared to the activity in the presence of the PS II-specific acceptors, improved rates of oxygen evolution were also observed for each of the double mutants lacking PsbM as well as the ∆PsbT:∆PsbU and ∆PsbT:∆PsbO mutants when bicarbonate was present (Table 1; Fig. 4).

**Table 1:** Maximum rates of oxygen evolution in wild-type *Synechocystis* sp. PCC 6803 and strains lacking PsbM or PsbT and the extrinsic proteins PsbO, PsbU and PsbV

Strains	Rate of O_2_ evolution (µmol (mg Chl)^−1^ h^− 1^)^a^								
	DMBQ			DCBQ			Bicarbonate		
wild type	438	±	14	525	±	05	497	±	41
∆PsbO	259	±	15	239	±	16	492	±	40
∆PsbU	356	±	46	349	±	08	467	±	49
∆PsbV	232	±	21	164	±	14	418	±	22
∆PsbM	424	±	08	529	±	19	482	±	17
∆PsbM:∆PsbO	195	±	07	160	±	25	416	±	50
∆PsbM:∆PsbU	316	±	35	403	±	39	473	±	60
∆PsbM:∆PsbV	154	±	05	140	±	07	407	±	12
∆PsbT	276	±	40	277	±	20	337	±	69
∆PsbT:∆PsbO	174	±	35	164	±	12	319	±	68
∆PsbT:∆PsbU	234	±	39	156	±	55	262	±	11
∆PsbT:∆PsbV^b^	0	±	n/a	0	±	n/a	0	±	n/a

^a^Rates of oxygen evolution are calculated for the initial period of actinic light illumination. Data are the average of three biological repeats with the standard error

^b^The strain was unable to evolve oxygen

n/a = not applicable

### The impact of removing the extrinsic proteins was also evident in the variable chlorophyll fluorescence transient from the mutants

**Fig. 5:**
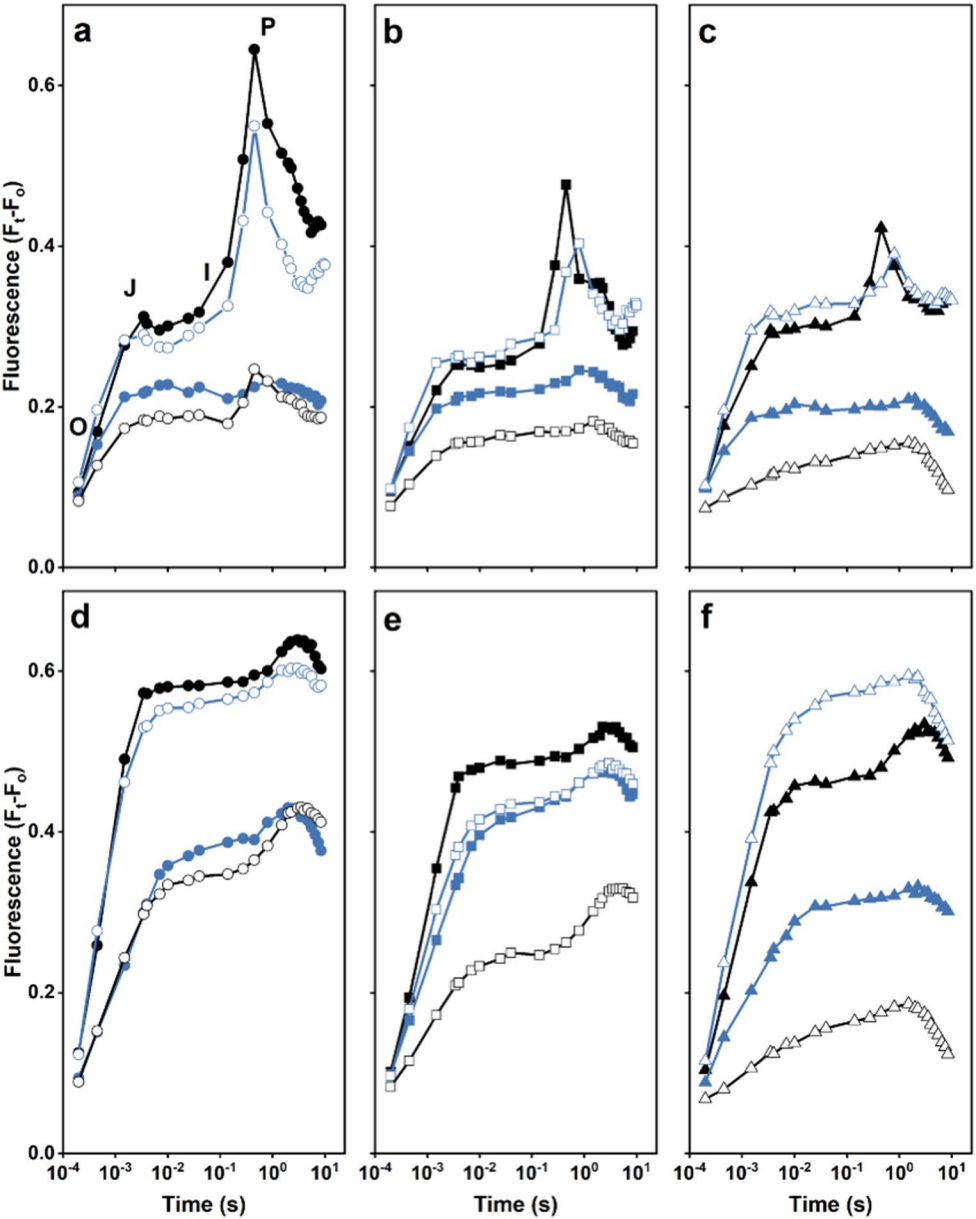
Chlorophyll *a* variable fluorescence induction. **a – c** No addition. **d – f** In the presence of DCMU. In panels **a** and **d** the traces are: wild type (black filled circles), ΔPsbO (blue filled circles), ΔPsbU (blue empty circles), ∆PsbV (black empty circles); in panels **b** and **e**: ΔPsbM (black filled squares), ΔPsbM:ΔPsbO (blue filled squares), ΔPsbM:ΔPsbU (blue empty squares), ΔPsbM:ΔPsbV (black empty squares), and in panels **c** and **f**: ΔPsbT (black filled triangles), ΔPsbT:ΔPsbO (blue filled triangles), ΔPsbT:ΔPsbU (blue empty triangles), ΔPsbT:ΔPsbV (black empty triangles). In panel **a**: O is the origin or initial fluorescence; and J is the first inflection point, I is the second inflection point and P is the peak of the fluorescence induction curve (additional details in text). F_t_, fluorescence at time t; F_o_, fluorescence in dark-adapted cells. Data are the average of three independent experiments

To further assess PS II activity and abundance in the different strains, variable chlorophyll *a* fluorescence induction was measured in the absence (Fig. 5a–c) and presence (Fig. 5d–e) of the PS II-specific herbicide, DCMU. In the absence of DCMU, wild-type cells exhibited a typical OJIP fluorescence induction curve (Fig. 5a): following the initial O-level the fluorescence increased to the J-level, due to Q_A_ reduction, followed by a rise from an inflection (I) to a maximum P level (or F_max_) as the available electron acceptors were reduced. The fluorescence then declined as a result of the activation of the Calvin-Benson cycle along with various quenching mechanisms (Stirbet and Govindjee 2011). In the ∆PsbU strain the P level was reduced and all strains lacking PsbO or PsbV lacked distinct OJIP features, consistent with there being few active PS II centers in these mutants (Summerfield et al. 2013). In contrast, the OJIP features were preserved in the ∆PsbM:∆PsbU and ∆PsbT:∆PsbU strains but a delay in reaching the P peak was evident in both mutants. Moreover, the J level was elevated in the ∆PsbT and ∆PsbT:∆PsbU strains indicative of impaired electron transfer between Q_A_ and Q_B_ (or Q_B_^**–**^) in these mutants.

In the presence of DCMU (Fig. 5d–f), electron transfer from Q_A_^**–**^ is blocked and the level of fluorescence is an indication of the relative level of active PS II centers present (Vass et al. 1999). Similar levels of PS II were detected in wild type and ∆PsbU cells and also in ∆PsbO and ∆PsbV cells, although the latter two mutants were reduced by ~ 50% compared with wild type (Fig. 5d). The ∆PsbM and ∆PsbT strains had similar but reduced variable fluorescence compared with wild type; however, the ∆PsbT:∆PsbU strain exhibited an increased fluorescence yield over that observed for the ∆PsbT strain (Fig. 5e, f). In addition, the variable fluorescence in the ∆PsbT:∆PsbO cells was below that observed for the ∆PsbM:∆PsbO cells with the fluorescence from the ∆PsbM:∆PsbO mutant remaining similar to that observed with the ∆PsbO strain (Fig. 5d, e). The ∆PsbM:∆PsbU mutant also exhibited a similar fluorescence induction to that obtained with the ∆PsbM:∆PsbO mutant. Furthermore, the ∆PsbT:∆PsbV strain had negligible variable fluorescence and the ∆PsbM:∆PsbV strain exhibited low variable fluorescence consistent with the relative number (or absence) of assembled centers detected in these mutants by BN-PAGE (cf. Figures 3 and 5d–f).

### Electron transfer probed through the decay of chlorophyll fluorescence following a single turnover actinic flash

**Fig. 6:**
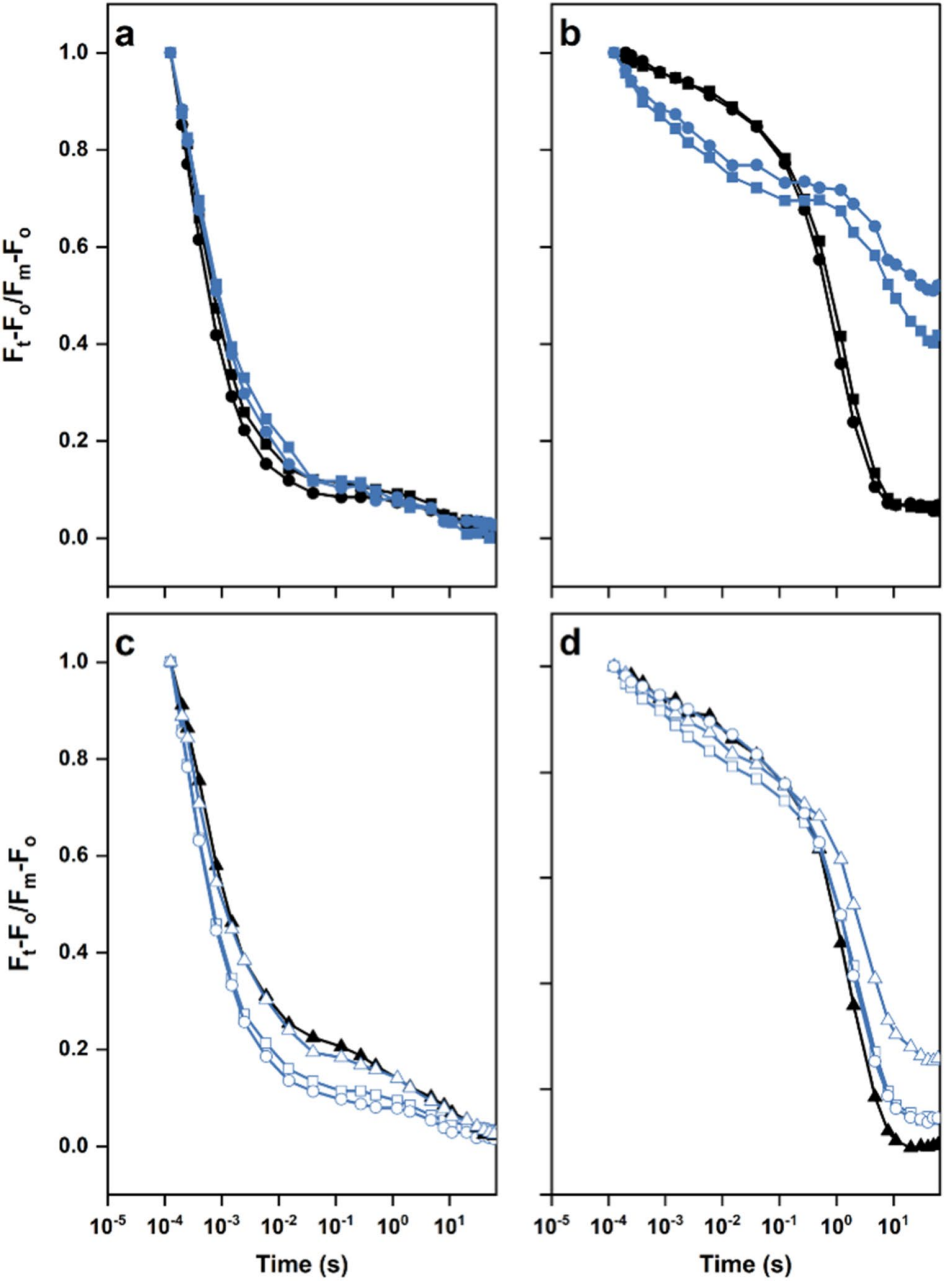
Decay of chlorophyll *a* fluorescence after a single actinic flash in the absence or presence of DCMU. **a** No addition: wild type (black filled circles), ∆PsbM (black filled squares), ∆PsbO (blue filled circles), ∆PsbM:∆PsbO (blue filled squares). **b** Strains and symbols are the same as panel **a** but in the presence of DCMU. **c** ∆PsbU (blue empty circles), ∆PsbT (black filled triangles), ∆PsbM:∆PsbU (blue empty squares) and ∆PsbT:∆PsbU (blue empty triangles). **d** Strains and symbols are the same as panel **c** but in the presence of DCMU. Fm, maximum fluorescence after actinic flash; F_t_, fluorescence at time t after actinic flash; F_o_, fluorescence in dark-adapted cells. Data are the average of three independent experiments

The different rates of oxygen evolution obtained with the mutants in the presence of the PS II- specific electron acceptors (Fig. 4; Table 1) suggested removal of the extrinsic proteins in the double mutants could further modify the acceptor side of PS II beyond any impact of removing PsbM or PsbT. Since the transfer of an electron between Q_A_ and Q_B_ can be followed by measuring the decay of variable fluorescence after a single turnover actinic flash, we selected the strains from Fig. 5 that exhibited sufficient variable fluorescence for this analysis. The data in Fig. 5 and Fig. S2 indicated that there were sufficient assembled PS II centers in the ∆PsbM:∆PsbO, ∆PsbM:∆PsbU, and ∆PsbT:∆PsbU strains, together with the corresponding single mutants, to complete a kinetic analysis (Fig. 6 and Tables S2 and S3). In the absence of DCMU, the fluorescence decay can be divided into three kinetically distinct components: a fast (µs) phase, ascribed to oxidation of Q_A_^**–**^ by Q_B_ (or Q_B_^**–**^ when present), an intermediate (ms) phase, ascribed to Q_B_ binding and subsequent oxidation of Q_A_^**–**^, and a slow (s) phase, ascribed to the back reaction of Q_A_^**–**^ with the S_2_-state of the OEC (Vass et al. 1999). In addition, in the presence of DCMU, a single saturating flash reduces all Q_A_ to Q_A_^**–**^ and the decay of fluorescence exhibits two phases: a ms phase, likely associated with recombination between Q_A_^**–**^ and the oxidized form of the redox-active tyrosine (D1-Tyr161 or TyrZ^•^) found between the OEC and P680 or between Q_A_^**–**^ and P680^+^; and a slow (s) component associated with recombination with S_2_ (Vass et al. 1999).

Consistent with previous reports, forward electron transfer was slowed in the ∆PsbT and ∆PsbM cells as well as in the ∆PsbO mutant (Biswas and Eaton-Rye 2008; Forsman and Eaton-Rye 2021; Choo et al. 2022). In contrast, there was little impact of removing PsbU by itself or in combination with PsbM in the ∆PsbM:∆PsbU strain (Table S2). Likewise the fluorescence decay in the ∆PsbT:∆PsbU double mutant was similar to that observed in the ∆PsbT strain (Fig. 6 and Table S2). In the presence of DCMU, however, removal of PsbO, both in the single mutant and in the ∆PsbM:∆PsbO strain, resulted in impairment of the back reaction with the fluorescence decaying by approximately 46% and 57%, respectively, over the period of measurement (Fig. 6b and Table S3). Removal of PsbT slowed the back reaction with the slow component extending from a t_1/2_ of 0.7 s (amp 90%) in wild type to a t_1/2_ of 2.1 s (amp 90%) in ∆PsbT cells. The additional removal of PsbU in the ∆PsbT:∆PsbU double mutant also impacted the back reaction with the ms component extending from a t_1/2_ of 1.7 ms (amp 10%) to a t_1/2_ of 3.9 ms (amp 15%) and the amplitude of the slow component reducing to 60% with the fluorescence not decaying fully back to zero over the course of the measurement (Fig. 6b and Table S3).

## Discussion

### Three-helix bundles formed by low-molecular-weight proteins in PS II

In *Synechocystis* sp. PCC 6803 (and in thermophilic cyanobacteria) the 13 LMW subunits are located at the periphery of the mature PS II complex (Shen 2015; Gisriel et al. 2021). It has been noted that a similar pattern of small membrane-spanning proteins is present in cyanobacterial and plant Photosystem 1 (PS I), suggesting the antecedents of these subunits were present in the ancestral photosystem that gave rise to PS I and PS II (Cardona 2016; and see Jordan et al. 2001; Mazor et al. 2014; Mazor et al. 2015). In particular, the three-helix bundle formed by PsbJ, PsbE and PsbF may have a counterpart in the three transmembrane helices of PsaL while PsbL, PsbM and PsbT are in a similar position to the three helices provided by PsaJ and PsaF (Cardona 2016). Since the LMW proteins are absent in the light-induced quinone reductase of anoxygenic photosynthetic bacteria it seems likely that the LMW proteins in PS II have been retained because they play a role in either enabling water splitting or in the repair cycle following photodamage (Shi et al. 2012; Vass 2012). All oxygenic photosynthetic organism also possess lumenal extrinsic proteins that play multiple roles in stabilizing and protecting the environment of the OEC and in providing channels for substrate access and the egress of O_2_ and protons (Ifuku and Nagao 2021; Hussein et al. 2023). We have considered if there are functional relationships between the PsbE/PsbF/PsbJ and PsbL/PsbM/PsbT three-helix bundles and the three extrinsic PsbO, PsbU and PsbV proteins that are common to the PS II structures available from *Synechocystis* sp. PCC 6803 and thermophilic cyanobacteria.

Cytochrome *b*_559_ (Cyt *b*_559_) composed of PsbE and PsbF, is required for formation of the D2 assembly module. The absence of either PsbE or PsbF therefore prevents PS II biogenesis since the reaction center pre-complex containing D1 and D2 cannot assemble (Komenda et al. 2004; Knoppová et al. 2022). In higher plants, however, it has been shown that Cyt *b*_559_ interacts with the extrinsic PsbP protein (Ido et al. 2012; Nishiyama et al. 2016). Since mutants lacking either PsbE or PsbF are not photoautotrophic our investigation of the PsbE/PsbF/PsbJ bundle was restricted to the characterization of the ∆PsbJ:∆PsbO, ∆PsbJ:∆PsbU and ∆PsbJ:∆PsbV double mutants (Choo et al. 2022). In *Synechocystis* sp. PCC 6803 photoautotrophic growth was slowed in the ∆PsbJ:∆PsbU strains and prevented altogether in ∆PsbJ:∆PsbO and ∆PsbJ:∆PsbV cells. However, PsbJ was able to bind to PS II in the absence of any one of the extrinsic proteins but an active OEC did not appear to assemble in the absence of PsbJ when either PsbO or PsbV were absent (Choo et al. 2022).

PsbJ is encoded in the *psbEFLJ* operon that is widely conserved among oxygenic photosynthetic organisms (Mor et al. 1995; Ifuku and Nagao 2021). Both the PsbJ and PsbL proteins influence forward electron transfer between Q_A_ and Q_B_ (Regel et al. 2001; Luo et al. 2014). Moreover, while PsbJ appears to bind to PS II upon attachment of the CP43 assembly module during biogenesis, the absence of PsbL severely retards this transition (Anbudurai and Pakrasi 1993; Bentley et al. 2008; Zabret et al. 2021). Hence, we focused on the impact of removing the extrinsic proteins in the absence of PsbM and PsbT.

### Removal of the extrinsic proteins in strains lacking PsbM

We constructed and characterized the six double mutants lacking either PsbM or PsbT and the PsbO, PsbU and PsbV proteins. The ∆PsbM:∆PsbO cells remained photoautotrophic and assembled a similar number of PS II monomers as those found in the ∆PsbO strain (Figs. 1a and b and 3a). However, photoautotrophic growth was slowed and ∆PsbM:∆PsbO cells had reduced rates of oxygen evolution compared to the ∆PsbO and ∆PsbU strains (Fig. 4; Table 1). The fluorescence decay following a single turnover actinic flash, however, did not reveal any large acceptor side effect in the double mutant beyond a slight increase in the slow component that may reflect a shift in the equilibrium for the sharing of the electron between Q_A_ and Q_B_ towards Q_A_^**–**^ (Fig. 6a; Table S2) (Vass et al. 1999). Consistent with these observations, fluorescence induction in the presence of DCMU, indicative of the number of photochemically active centers present, was similar between ∆PsbO and ∆PsbM:∆PsbO cells; however, upon 440 nm excitation, low-temperature fluorescence spectra gave slightly elevated 685 nm emission in the double mutant, but this is likely from unassembled CP43 protein (Fig. 3a). These results contrast with the ∆PsbJ:∆PsbO cells which were not photoautotrophic and did not evolve oxygen (Choo et al. 2022).

The removal of PsbV in the ∆PsbM background resulted in slower photoautotrophic growth, and reduced rates of oxygen evolution, than observed in the ∆PsbM:∆PsbO strain; however, this may in part be due to destabilized PsbO binding in this mutant (Fig. 3d). In addition, fewer photochemically active PS II centers were detected in the ∆PsbM:∆PsbV strain than in ∆PsbM:∆PsbO cells (Fig. 5) but PS II dimers were present (Fig. 3c). Low temperature fluorescence spectroscopy obtained with 440 nm excitation, however, exhibited a lower emission at 685 nm in the absence of PsbV than PsbO in the double mutants (Fig. 2b): the emission at 685 nm contains contributions from CP43 in assembled PS II, and CP43 and CP47 in unassembled PS II, and it may be influenced by the extent of dimerization (Boehm et al. 2011). Consistent with the fluorescence induction results, the low temperature emission spectra obtained with 580 nm excitation indicated that fewer centers were present for energy transfer from the phycobilisome in ∆PsbM:∆PsbV cells than in the ∆PsbM:∆PsbO strain (Fig. 2e). As expected from the photoautotrophic growth curve, the centers present in the ∆PsbM:∆PsbV mutant were able to support oxygen evolution in the presence of bicarbonate (employing the native quinone) although lower rates were obtained with DCBQ and DMBQ (Table 1). When compared, PS II in the ∆PsbM:∆PsbV cells appeared less severely affected than the in the ∆PsbJ:∆PsbV strain.

Lastly, the ∆PsbM:∆PsbU strain was less impacted across all assays than the ∆PsbM:∆PsbO mutant. Typically, removal of PsbU results in a milder deleterious phenotype than removal of either PsbO or PsbV across a wide range of conditions (reviewed in: Bricker et al. 2012; Roose et al. 2016). Furthermore, it has previously been documented that the absence of PsbU leads to uncoupling of energy transfer from the phycobilisome into PS II in cells possessing an additional mutation in the ChlH subunit of magnesium chelatase (Veerman et al. 2005; Morris et al. 2014; Crawford et al. 2016). Since the low-temperature fluorescence spectra obtained with 580 nm excitation for the ∆PsbJ:∆PsbU, ∆PsbM:∆PsbU, and ∆PsbT:∆PsbU strains all indicate normal energy transfer between the phycobilisome and PS II, these data point to a specific functional connection between the role of PsbU in stabilizing PS II and the availability of chlorophyll during PS II biogenesis (Roose et al. 2016; Choo et al. 2022).

### Removal of the extrinsic proteins in strains lacking PsbT

Photoautotrophic growth and rates of oxygen evolution upon removal of the extrinsic proteins in the absence of PsbT followed the expected order of severity with ∆PsbT:∆PsbU cells the least affected and ∆PsbT:∆PsbO cells displaying an intermediate but still photoautotrophic phenotype. In contrast, the ∆PsbT:∆PsbV mutant was not photoautotrophic and could not evolve oxygen (Figs. 1 and 4; Table 1). Notably in the ∆PsbT:∆PsbU mutant, the PS II-specific peaks at 685 and 695 nm in the low temperature fluorescence emission spectrum upon excitation at 440 nm were elevated (Fig. 2c). In addition, fluorescence induction in the presence of DCMU was also unexpectedly high from the ∆PsbT:∆PsbU cells (Fig. 5f). These increased fluorescence signals are not explained by the presence of unassembled CP43-containing complexes observed in this mutant (Fig. 3b). However, similar increased fluorescence signals have been observed in mutants carrying substitutions at D1-Glu244 and D1-Tyr246 that are associated with the bicarbonate-binding environment on the acceptor side of PS II (Forsman et al. 2019). Moreover, similar fluorescence signals have been observed in strains with mutations at D1-His252 that participates in proton delivery to Q_B_^**–**^ (Forsman et al. 2024). These D1 mutants are in the large DE-loop of the D1 protein, and it has also been shown that PsbT directly interacts with this loop (Forsman and Eaton-Rye 2021). In the D1 mutants the elevated fluorescence has been shown to be a blue-light specific effect that is observed in intact cells but not in isolated thylakoids (Forsman et al. 2024). It is therefore noteworthy that the low temperature emission spectrum for ∆PsbT:∆PsbU cells upon excitation at 580 nm did not show any enhanced emission at 685 nm; which is also consistent with their being similar levels of assembled centers in both ∆PsbT and ∆PsbT:∆PsbU cells (Figs. 2f and 3a and b). The molecular mechanism of this effect has not yet been uncovered but it has been suggested that energy transfer into PS II in these mutants may involve energy spillover that requires a supercomplex that dissociates upon thylakoid extraction (Forsman et al. 2024; Sheridan et al. 2024).

Among the six double mutants investigated, the ∆PsbT:∆PsbV strain was the most impacted and exhibited a phenotype that was as severe as that of the ∆PsbJ:∆PsbV mutant (Choo et al. 2022). The ∆PsbT:∆PsbV mutant did not assemble stable dimers, potentially due to destabilized PsbO binding, and an enhanced presence of the RC47 complex was apparent in this strain (Fig. 3c, d). It is likely that the absence of any signal for PsbO in Fig. 3d is the result of unbound PsbO in the thylakoid lumen being lost during membrane isolation. Furthermore, the reduced PS II level in these cells was reflected in the low-temperature fluorescence emission spectrum with excitation at 580 nm with the energy from the phycobilisome terminal emitter being released at 685 nm (Fig. 2f).

It has previously been shown that the ∆PsbO:∆PsbV double deletion mutant is not photoautotrophic and only supports oxygen evolution at approximately 10% of the wild-type rate (Shen et al. 1995); hence the absence of PsbT, in combination with the absence of PsbV, may destabilize PsbO binding to the extent that the ∆PsbT:∆PsbV cells also lack PsbO (and quite possibly PsbU) and that this is responsible for their inability to grow photoautotrophically or to evolve oxygen. Our data in Fig. 3d also suggest that PsbO binding may be weakened in the ∆PsbM:∆PsbV strain, contributing to the phenotype observed with these cells; however, unlike the ∆PsbT:∆PsbV cells, the ∆PsbM:∆PsbV mutant was able to support oxygen evolution at approximately 84% of the wild-type rate when the native quinone was operating in whole chain electron transport in the presence of bicarbonate. Hence, PsbT appears to make a stronger contribution to PsbO binding in the absence of PsbV than PsbM.

## Conclusion

Neither PsbM nor PsbT have direct protein-protein interactions with the extrinsic proteins; however, these LMW proteins, found at the monomer-monomer interface of the mature PS II complex, have synergistic roles with PsbO, PsbU and PsbV. The removal of the extrinsic proteins in cells lacking PsbT produced more disruption to PS II assembly and activity than their removal in the absence of PsbM. The extent of the observed perturbation of PS II was most evident when the PsbV protein was removed and least evident when PsbU was absent. However, in ∆PsbT:∆PsbU cells the variable fluorescence from PS II and low-temperature emission from PS II was elevated despite their being no accompanying increase in the amount of PS II detected. Among the six mutants examined only the ∆PsbT:∆PsbV mutant was unable to grow photoautotrophically or to evolve oxygen. Since PS II monomers were present in the ∆PsbT:∆PsbV cells, the results suggest the OEC cannot assemble in this double mutant.

### Electronic supplementary material

Below is the link to the electronic supplementary material.

Supplementary Material 1

## Data Availability

The data used to generate the figures in this manuscript are available on request by emailing the corresponding author.
