# Supplementary material for: Indirect interactions involving the PsbM or PsbT subunits and the PsbO, PsbU and PsbV proteins stabilize assembly and activity of Photosystem II in Synechocystis sp. PCC 6803
Source: Photosynth Res. 2024 Mar 15;160(2-3):61–75. doi: 10.1007/s11120-024-01091-9 (PMC11108944; doi:10.1007/s11120-024-01091-9)
Supplement: Supplementary file 1 — Supplementary Material 1 [file 11120_2024_1091_MOESM1_ESM.pdf]

Supplementary Information

Indirect interactions involving the PsbM or PsbT subunits and the PsbO, PsbU and PsbV proteins stabilize assembly and activity of Photosystem II in *Synechocystis* sp. PCC 6803

Faiza Arshad[1] • Julian J. Eaton-Rye[1]

[1]Department of Biochemistry, University of Otago, Dunedin, New Zealand

Corresponding Author Julian J. Eaton-Rye
julian.eaton-rye@otago.ac.nz

Contents

Page 3 **Fig. S1** Agarose gels showing the segregation of the introduced gene deletions or interruptions in the different mutants using the primers in Table S1.

Page 4 **Fig. S2** Decay of chlorophyll *a* fluorescence after a single actinic flash in the absence or presence of DCMU.

Page 5 **Table S1** List of primers used in the study.

Page 6 **Table S2** Decay kinetics of flash-induced variable fluorescence after a single turnover flash.

Page 7 **Table S3** Decay kinetics of flash-induced variable fluorescence after a single turnover flash in the presence of 40 µM DCMU.

Page 8 **References**

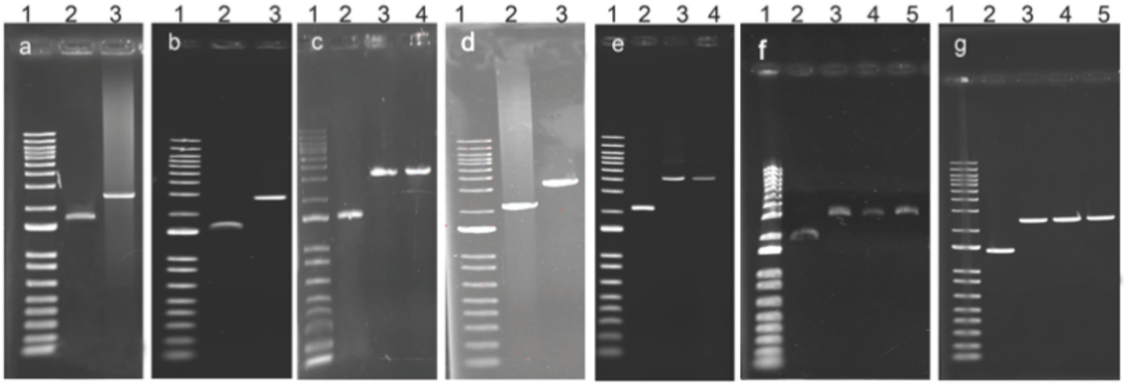

Fig. S1 Agarose gels showing the segregation of the introduced gene deletions or interruptions in the different mutants using the primers in Table S1. **a** Lanes are: (1) 1 kb Plus ladder (Invitrogen, Carlsbad, CA, U.S.A.); (2) *psbT* gene in wild type; (3) *psbT* replaced with a chloramphenicol-resistance cassette. **b** Lanes are: (1) 1 kb Plus ladder (Invitrogen); (2) *psbM* gene in wild type; (3) the *psbM* gene knocked out by insertion of a kanamycin-resistance cassette. **c** Lanes are: (1) 1 kb Plus ladder (Invitrogen); (2) *psbM* gene in wild type; (3) *psbM* gene knocked out by insertion of a spectinomycin-resistance cassette in ΔPsbM:ΔPsbV cells; (4) *psbM* gene knocked out by insertion of the spectinomycin-resistance cassette in ΔPsbM:ΔPsbU cells. **d** Lanes are: (1) 1 kb Plus ladder (Invitrogen); (2) *psbO* gene in wild type; (3) *psbO* replaced with a kanamycin-resistance cassette. **e** Lanes are: (1) 1 kb Plus ladder (Invitrogen); (2) *psbO* gene in wild type; (3) *psbO* gene knocked out by insertion of a spectinomycin-resistance cassette in ΔPsbM:ΔPsbO cells; (4) *psbO* gene knocked out by insertion of the spectinomycin-resistance cassette in ΔPsbT:ΔPsbO cells. **f** Lanes are: (1) 1 kb Plus ladder (Invitrogen); (2) *psbV* gene in wild type; (3) *psbV* gene knocked out by insertion of a kanamycin-resistance cassette; (4) *psbV* gene knocked out by insertion of a kanamycin-resistance cassette in ΔPsbM:ΔPsbV cells; (5) *psbV* gene knocked out by insertion of the kanamycin-resistance cassette in ΔPsbT:ΔPsbV cells. **g** Lanes are: (1) 1 kb Plus ladder (Invitrogen); (2) *psbU* gene in wild type; (3) *psbU* gene knocked out by insertion of a kanamycin-resistance cassette; (4) *psbU* gene knocked out by insertion of a kanamycin-resistance cassette in ΔPsbM:ΔPsbU cells; (5) *psbU* gene knocked out by insertion of the kanamycin-resistance cassette in ΔPsbT:ΔPsbU cells. The insertion sites of the antibiotic-resistance cassettes were confirmed by Sanger sequencing and the details are provided in the Methods section.

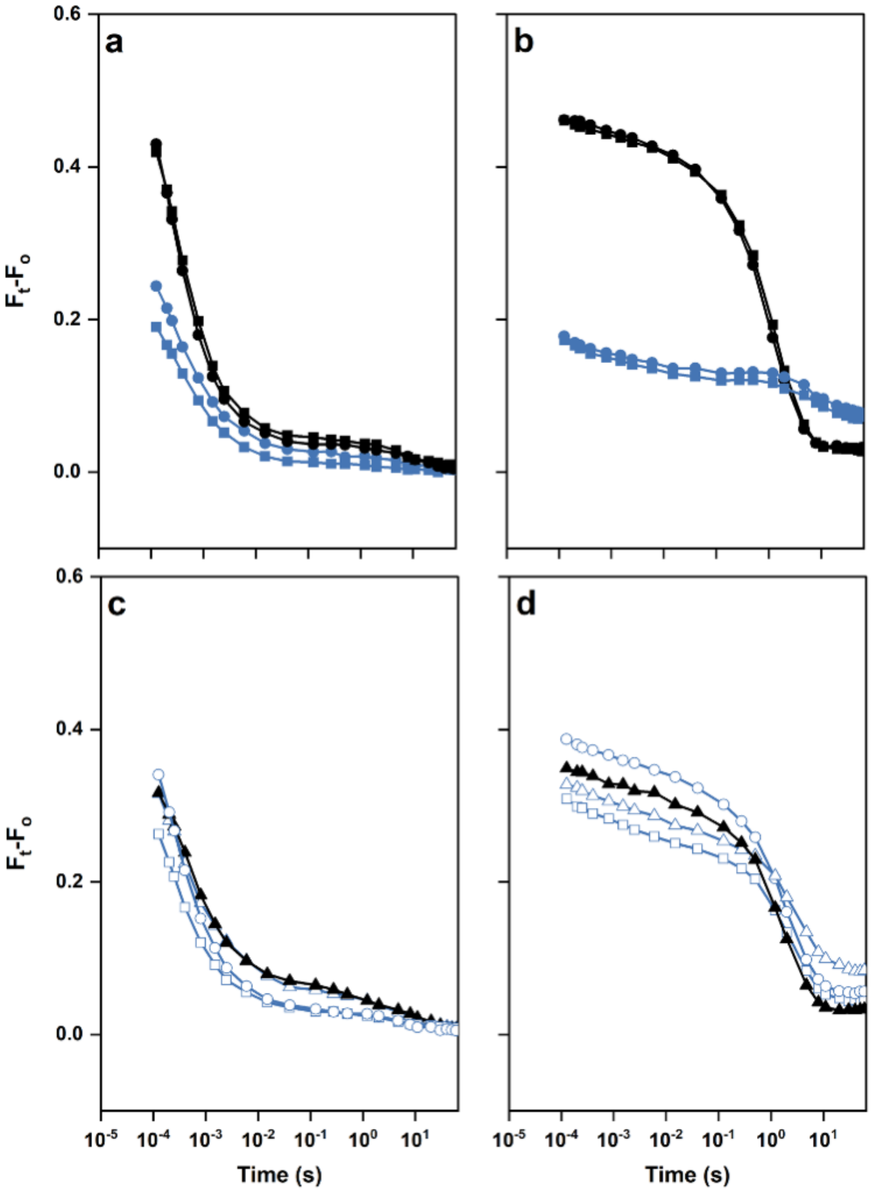

Fig. S2 Decay of chlorophyll *a* fluorescence after a single actinic flash in the absence or presence of DCMU. **a** No addition: wild type (black filled circles), ΔPsbM (black filled squares), ΔPsbO (blue filled circles), ΔPsbM:ΔPsbO (blue filled squares). **b** Strains and symbols are the same as panel a but in the presence of DCMU. **c** ΔPsbU (blue empty circles), ΔPsbT (black filled triangles), ΔPsbM:ΔPsbU (blue empty squares) and ΔPsbT:ΔPsbU (blue empty triangles). **d** Strains and symbols are the same as panel c but in the presence of DCMU. F_t, fluorescence at time t after actinic flash; F_o, fluorescence in dark-adapted cells. Data are the average of three independent experiments.

Table S1 List of primers used in the study

Oligo name	Oligo sequence
PsbO Forward	5’CTGCTCCAGGCTTTCCTGCA3’
PsbO Reverse	5’ACGCCGAACTGAGGAAGAGCA3’
PsbU Forward	5’CGGGTTTTGATCAGTCAGTCATTTGG3’
PsbU Reverse	5’GCCACACCTGTCAGTATTGC3’
PsbV Forward	5’GCAACCAACAGATAGGCAGTGGAC3’
PsbV Reverse	5’CCGAAGAGAAGTTGCAGTATTGCG3’
PsbM Forward	5’CAGGGGGACGGGCCAACACAAACAC3’
PsbM Reverse	5’CGGCGGTGCAAAAGGCATCGATCAC3’
PsbT Forward	5’CGGTTGAGCATTGACCCGATGGCTG3’
PsbT Reverse	5’TTCGTGATGACGGTAACCTCTGCCG3’

Table S2 Decay kinetics of flash-induced variable fluorescence after a single turnover flash[a,b]

	Fast Component		Intermediate Component		Slow Component	
Strain	Rate ($t_{1/2}$ = μs)	Amplitude (%)	Rate ($t_{1/2}$ = ms)	Amplitude (%)	Rate ($t_{1/2}$ = s)	Amplitude (%)
wild type	241 ±13	67.1 ±5.1	2.6 ±0.1	21.9 ±1.7	6.7 ±11	11.0 ±0.5
ΔPsbT	422 ±38	62.6 ±2.7	11.0 ±1.2	24.0 ±0.9	7.0 ±1.1	13.4 ±1.3
ΔPsbM	301 ±10	62.5 ±4.8	5.1 ±0.3	25.6 ±1.2	8.2 ±0.5	11.9 ±1.2
ΔPsbO	321 ±14	61.5 ±0.5	6.2 ±1.1	27.5 ±1.2	11.4 ±9.2	11.5 ±0.7
ΔPsbM:ΔPsbO	373 ±23	60.3 ±0.3	4.4 ±1.2	28.2 ±0.4	4.7 ±0.8	11.5 ±0.3
ΔPsbU	264 ±16	67.7 ±5.3	3.9 ±0.1	22.5 ±0.1	8.5 ±1.5	9.8 ±0.1
ΔPsbM:ΔPsbU	278 ±17	59.5 ±10.1	5.1 ±0.5	26.4 ±0.7	15.4 ±0.1	14.1 ±0.1
ΔPsbT:ΔPsbU	328 ±11	63.0 ±15.1	8.3 ±2.1	25.1 ±1.8	9.0 ±2.1	11.9 ±0.3

[a]Data analyzed according to Vass et al. (1999). The fast (μs) component represents forward electron transfer from Q_A^- to Q_B or, if present, Q_B^-. The intermediate (ms) component represents electron transfer from Q_A^- when the Q_B-binding site is empty and a Q_B from the plastoquinone pool must first bind to the vacant site before the electron is transferred. The slow (s) component represents the back reaction from Q_A^- to the Mn_4CaO_5 cluster in the S2 state. The kinetics of the first two phases are exponential functions, whereas the slow phase is described by a hyperbolic function. Following Vass et al. (1999) the decay curve was therefore deconvoluted using the equation $F_{(t)} - F_o = A_1\exp^{(-t/T1)} + A_2\exp^{(-t/T2)} + A_3/(1 + t/T3)$; where, $F_{(t)}$ is the yield of variable fluorescence, F_o is the initial fluorescence from dark-adapted cells, A_1, A_2, A_3 are the amplitudes and T_1, T_2, T_3 are the time constants. The T_1 and T_2 values are used to calculate the half-life of the fast and intermediate phases by using the equation $t_{1/2} = \ln 2\ T$, whereas the half-life for hyperbolic phase is equal to T_3 (Vass et al. 1999).

[b]The standard error is calculated from three biological repeats.

Table S3 Decay kinetics of flash-induced variable fluorescence after a single turnover flash in the presence of 40 µM DCMU[a,b]

Strain	Milliseconds Component		Seconds Component	
	Rate ($t_{1/2}$ = ms)	Amplitude (%)	Rate ($t_{1/2}$ = s)	Amplitude[c] (%)
wild type	1.4 ±0.2	9.8 ±1.3	0.7 ±0.1	90.2 ±0.6
ΔPsbT	1.7 ±0.2	10.2 ±0.1	2.1 ±0.1	89.8 ±0.2
ΔPsbM	2.3 ±0.2	8.1 ±0.1	0.8 ±0.1	87.1 ±0.3
ΔPsbO	2.5 ±0.2	18.9 ±0.5	5.5 ±1.1	27.1 ±0.3
ΔPsbM:ΔPsbO	2.5 ±0.5	21.7 ±0.1	6.1 ±1.3	35.1 ±1.3
ΔPsbU	3.5 ±0.4	11.2 ±0.2	1.3 ±0.1	75.1 ±0.1
ΔPsbM: ΔPsbU	1.6 ±0.2	15.4 ±0.1	1.5 ±0.1	70.4 ±0.7
ΔPsbT: ΔPsbU	3.9 ±1.1	14.8 ±0.1	2.2 ±0.1	59.9 ±0.1

[a]In the presence of DCMU, the ms exponential component likely reflects recombination with $TyrZ^{\bullet}$ (or $P680^{+}$ if the Mn_4CaO_5 cluster is impaired) and the slow (s) hyperbolic component reflects recombination with the S_2 state of the Mn_4CaO_5 cluster (Vass et al., 1999; Forsman and Eaton-Rye, 2021).

[b]The standard error is calculated from three biological repeats.

[c]The sum of amplitudes is not equal to 100% for the mutants where the chlorophyll fluorescence was unable to decay back to 0 in 60 s.

References

Forsman JA, Eaton-Rye JJ (2020) The Interaction between PsbT and the DE Loop of D1 in Photosystem II stabilizes the quinone–iron electron acceptor complex. Biochemistry 60:53–63. https://doi.org/10.1021/acs.biochem.0c00668

Vass I, Kirilovsky D, Etienne A (1999) UV-B radiation-induced donor- and acceptor-side modifications of Photosystem II in the cyanobacterium *Synechocystis* sp. PCC 6803. Biochemistry 38:12786–12794. https://doi.org/10.1021/bi991094w
